# A comparative scoping review approach: identifying the intersection of carbon, biodiversity, and water offsetting

**DOI:** 10.1016/j.mex.2026.103799

**Published:** 2026-01-13

**Authors:** Felice Diekel, Rosalie Arendt, Markus Berger

**Affiliations:** University of Twente, Multidisciplinary Water Management, Faculty of Engineering Technology, Department of Civil Engineering & Management, Enschede, the Netherlands

**Keywords:** Scoping review, Comparative scoping review, Environmental policy, Climate policy, Offsetting, Carbon, Biodiversity, Water

## Abstract

Environmental and climate policies, as well as the knowledge underpinning them, are often developed in isolation. This is evident in offsetting research and policy, which tend to address carbon, biodiversity, and water as separate issues. This paper presents the development of an adapted scoping review methodology to compare these three distinct bodies of literature within a unified framework, which also allows for the introduction of the emerging water offsetting literature. The approach ensures comparability across datasets of relevant literature while addressing the challenge of managing large volumes of literature within time and resource constraints. It provides a practical solution for managing diverse bodies of literature in scoping reviews, enabling a holistic understanding of the interrelationships among carbon, biodiversity, and water offsetting.

Key elements of the method include:

• Applying a consistent approach across all three datasets, while accommodating the specificities of each.
• Utilizing the machine learning tool ASReviewer to streamline the screening process, alongside a pilot screening phase to establish consistent inclusion criteria.
• Combining quantitative bibliometric analysis with qualitative thematic analysis.

## Graphical abstract

**Figure:**
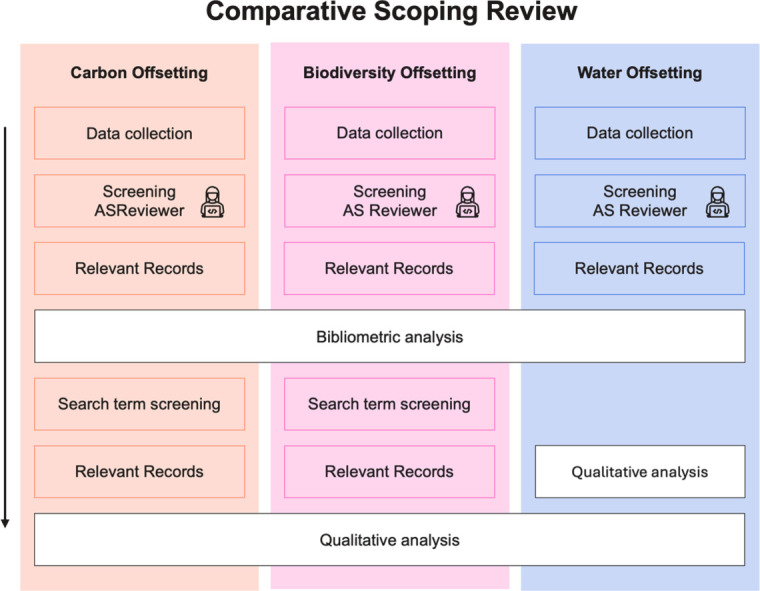

**Specifications table**

**Table:** 

Subject area	Environmental Science
More specific subject area	Environmental and climate policy
Name of your method	Comparative scoping review
Name and reference of original method	Scoping review
Resource availability	ASReviewer: https://asreview.nl/

## Background

Both policy-making and knowledge production often suffer from siloed development and limited communication, leading to missed synergies, overlooked co-benefits, and unintended burden-shifting or dis-benefits [1]. A clear example of this fragmentation is found in offsetting policies, which span multiple policy domains, particularly climate and environmental policy, as well as distinct strands of academic literature [2].

Building on the emerging practical use of intersecting offsetting types (e.g. carbon, biodiversity, and water) and high level calls for better integration (e.g., IPBES and IPCC workshop [3]), we analyzed a potential intersection of offsetting types in the scientific literature in the original research article associated with this MethodsX paper (related research article). Our study identified existing divides, research gaps, and the rare instances of integration across carbon, biodiversity, and water offsetting. By systematically comparing these offsetting types within a single study and introducing water offsetting into the analysis, we offer a novel contribution, as previous research typically focuses on only one type or explored different combinations using less comprehensive or less structures approaches [2,4]. Integrating these three offsetting approaches offers a comprehensive perspective, enabling bibliometric and qualitative comparisons while situating water offsetting within the broader offsetting literature. This not only contextualizes water offsetting within existing research and policy discussions but also reveals connections, distinctions, and overarching patterns that might otherwise remain unnoticed. By adapting a scoping review approach to synthesize insights across these distinct yet interrelated fields, this study lays a foundation for future research at their intersection, fostering a more integrated and cohesive exploration of offsetting mechanisms in environmental policy.

However, conducting a comparative scoping review across these three offsetting types presents significant challenges. Carbon and biodiversity offsetting each have well-established research traditions. A scoping review of just one field is already time- and resource-intensive; addressing all three requires conducting multiple reviews simultaneously. This makes it crucial to integrate time- and resource-saving approaches while maintaining rigor. Additionally, defining a clear focus is essential to ensure the review remains comprehensive yet manageable.

Our approach is described in more detail in this paper as it can be of relevance beyond our study and can be adopted and adjusted by others to bring together distinct but interrelated bodies of literature, providing a structured way to compare and integrate them. By identifying common themes, gaps, and points of divergence, it supports more comprehensive analyses across fragmented research areas. Additionally, this method allows for the introduction of less-established bodies of literature into existing fields, helping to contextualize and compare emerging topics with more developed research. This makes it a valuable tool for interdisciplinary studies aiming to bridge knowledge gaps and foster integration in environmental and climate policy and beyond. Potential future research directions for this method could include extending the dataset to incorporate nutrient or nature offsetting, conducting a comparative scoping review of values related to water and biodiversity, and broadening the scope beyond an environmental focus to compare sustainability strategies for sectors such as transport, energy, and housing.

## Method details

For the specific purpose of integrating and comparing different bodies of literature, a scoping review approach was chosen as the foundation. A scoping review facilitates a "preliminary assessment of the potential size and scope of available research literature" ([5], p. 95) and is particularly well-suited for exploring and defining the conceptual and logistical boundaries of a topic through a systematic, transparent, and replicable process [5,6]. To enable a robust comparative analysis, the method was adapted to simultaneously analyze the three distinct literature sets, integrating bibliometric analysis and two qualitative approaches.

In developing the method, it was important to consider both the varying volumes and maturity of the three bodies of literature, as well as how these influenced the intended analysis. Carbon and biodiversity offsetting are well-established fields with extensive research histories, meaning that the primary aim for these two datasets was to make them comparable and to identify areas where they intersect. Consequently, throughout the method, both carbon and biodiversity offsetting datasets were treated identically to facilitate direct comparison. The water offsetting literature, on the other hand, is still under development, and a key part of this review was to introduce this emerging offsetting type into the broader literature. For this reason, the approach for the water offsetting literature deviated from the common approach applied to the other two datasets. A separate qualitative analysis was introduced for water offsetting to allow for a deeper exploration of its role, providing a way to integrate it into the literature and the intersection of the other two established bodies of knowledge. These deviations from the standard approach are highlighted and described at every step of the method to ensure clarity and transparency. To enhance overall consistency across datasets and over time, an activity journal was maintained throughout the screening process. This journal systematically documents each step and decision made, ensuring that if a step needs to be replicated for a subsequent dataset, it can be directly compared. Additionally, any deviations can be clearly identified, reported, and justified.

**Fig. 1:**
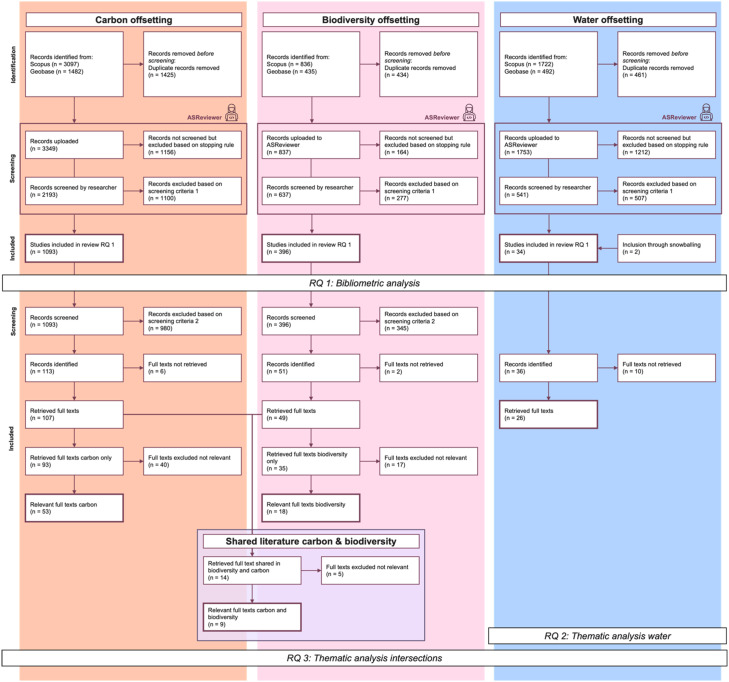
adapted PRISMA flowchart illustrating the review process (based on [18]).

The resulting adapted review process is visually represented in Fig. 1 as a revised PRISMA flowchart and is described in detail in the following sub-chapters.

## Data collection

The data collection was performed in the same manner for all three datasets, with the only deviation being the formulation of the search string for carbon offsetting. As relevant literature was expected from a variety of disciplinary backgrounds, Scopus was selected as the primary database due to its extensive coverage of interdisciplinary peer-reviewed literature [7], while Geobase as a disciplinary database was used to triangulate the results from Scopus. For both databases, titles, abstracts, and keywords were searched without a lower time limit, covering all available research up to February 9, 2024. The search included articles, reviews, book chapters, and conference papers, while grey literature was excluded due to the study’s scope and the large volume of scientific publications. The language was restricted to English and German, reflecting the research team’s language proficiency. No other filters were applied.

The search strings were developed by the research team in consultation with information specialists from the University of Twente and customized for each database. A common approach was used to generate search strings, ensuring comparability across the three offsetting types while allowing for adjustments based on the scope and maturity of each field. The core logic was to combine each offsetting type with related concepts such as no-net loss or neutrality. Given the vast body of literature on carbon offsetting, a more restrictive search strategy was applied, requiring these concepts to be directly linked to compensation or offsetting to maintain focus and relevance. The final search strings are presented in Table 1.

**Table 1:** Search strings.

Dataset	Database	Search string
Biodiversity	Scopus	TITLE-ABS-KEY ((biodiversity W/1 offset*) OR (biodiversity W/1 compensat*) OR (biodiversity AND (net-loss OR net-gain OR habitat-bank OR species-bank OR nature-positive)))
	Geobase	(((biodiversity NEAR/1 offset) OR (biodiversity NEAR/1 compensat) OR (biodiversity AND ({net loss} OR {net gain} OR {habitat bank} OR {species bank} OR {nature positive}))) WN KY)
Carbon	Scopus	TITLE-ABS-KEY ((carbon W/1 offset*) OR (carbon W/1 compensat*) OR ((offset* OR compensat*) AND (carbon-neutral OR net-zero OR carbon-negative OR climate-positive)))
	Geobase	(((carbon NEAR/1 offset) OR (carbon NEAR/1 compensate) OR ((offset OR compensate) AND ({carbon neutral} OR {net zero} OR {carbon negative} OR {climate positive}))) WN KY)
Water	Scopus	TITLE-ABS-KEY ((water W/1 offset*) OR (water W/1 compensat*) OR "water-positive" OR "water-neutr*")
	Geobase	((((water NEAR/1 offset) OR (water NEAR/1 compensat) OR {water positive} OR {water neutral})) WN KY)

## Screening

Screening preparations and the screening process were conducted consistently across all three datasets, except for adjustments made to the stopping rule and screening criteria for the water offsetting dataset, as explained below.

The data preparation process involved merging the Scopus and GeoBase files for each offsetting type, followed by deduplication, resulting in three distinct datasets ready for screening.

The screening process has been identified as a major bottleneck in reviews, being one of the most labor-intensive tasks [8]. To address this challenge we adopted two key strategies to efficiently manage the large volume of literature and address time and resource constraints across all three datasets: the use of a machine learning tool to streamline the screening process and a pilot screening phase to develop the screening criteria.

Establishing clear and consistent screening criteria is essential for ensuring the reliability of scoping reviews. While dual independent screening of all records by a team of researchers is considered best practice, resource and time constraints often make this unfeasible. To ensure consistency across the three datasets, we implemented a pilot screening phase to develop and refine the screening criteria before full screening.

In this pilot screening phase, two researchers independently screened 20 randomly selected records from each dataset, assessing their relevance based on title and abstract and providing a justification for their decision. The results were then compared, and discrepancies were discussed to harmonize the screening approach. Based on this pilot screening process the inclusion criteria were formulated to identify the most relevant records in a full screening process. An overview of the screening criteria (screening criteria 1) is provided in Table 2. For the water-related dataset**,** an adaptation was necessary, as none of the initial 20 randomly selected records were marked relevant, by either researcher. To address this, a non-random, purposeful additional round of pilot screening was conducted, including known relevant records to refine the criteria effectively. This structured pilot screening process improved consistency and transparency in the formulation of inclusion criteria while optimizing the use of available resources. The finalized screening criteria were then applied to the full datasets. During the screening process, refinements and additions to the criteria were made as necessary.

**Table 2:** Overview screening criteria 1.

Criteria	Description
Explicit focus on offsetting	Includes explicit focus on carbon, biodiversity, and/or water offsetting concepts, policies, or applications.
Exception for water literature	Given the limited number of publications in the water-related literature, topics such as water neutrality are included even when offsetting is not explicitly mentioned.
Understanding offsetting	The challenge lies in differentiating the intended use of "offsetting" as a policy tool or mechanism from its literal meaning. To make this distinction, we reference the definition in the original publication (related research article): (1) matching a benefit, (2) to a specific harm, (3) in an offsetting site distinct from the impact site, (4) following agreed-upon criteria. Publications that focus on offsetting as a policy tool are considered relevant, while those using the term in its literal sense (e.g., general balancing/replacing) are excluded. This includes cases where "offsetting" refers to practices like using stormwater instead of potable water or replacing surface water use with municipal groundwater pumping, which are considered replacements rather than offsetting within a conceptual meaning.
Implicit focus on offsetting	If offsetting is not explicitly named, related concepts such as "No Net Loss" (NNL) must clearly imply offsetting as a central topic. Discussions of conservation, restoration, forest growth rates, carbon sequestration, blue carbon projects, or general "nature-positive" concepts without explicit links to offsetting are excluded. Similarly, papers focused on the mitigation hierarchy, where offsetting is not central, are excluded.
(Non-offsetting) compensation mechanisms	Broad policy frameworks and compensation mechanisms, such as ecological compensation schemes, water compensation approaches, water rights trading, and water diversion project compensation are excluded. These schemes often involve financial compensation or ecosystem service payments, but their specific relation to offsetting can be ambiguous. Due to this uncertainty, they were excluded from the review.
Prominence of offsetting	Offsetting must play a significant role in the paper (at least 1/3 of the content), unless the paper is a review or policy analysis. If multiple topics are listed, the paper must include no more than two other main topics to be considered relevant. If more than three topics are listed, the paper is excluded. Additionally, if offsetting is only mentioned in the discussion section, particularly to discuss the results, it is not considered the main focus of the paper and is therefore excluded.
Methodology mentioning offsetting	If a paper introduces a tool, methodology, or similar, and only in the final sentence mentions that it could be relevant for offsetting, it is not considered relevant.
New forms of offsetting	If a new form of offsetting (e.g., socio-cultural offsetting) is introduced, it must be explicitly related to carbon, biodiversity, or water offsetting within the abstract to be considered relevant. Example: A paper discussing sport as a cultural offset was excluded because it had no relation to biodiversity, carbon, or water offsetting in the abstract.
Insetting	Insetting, such as carbon footprint offsetting within system boundaries (e.g., a grape farm offsetting its own carbon footprint through its own carbon sequestration efforts), is not considered relevant for inclusion.
Carbon balance and background carbon cycle	Offsetting activities that account for the carbon balance of the unmanaged or background carbon cycle are not considered relevant. For example, natural carbon uptake by the ocean or general national-level carbon sequestration (e.g., through forests) is not relevant, as these are part of the natural carbon cycle. Relevant offsetting must involve additional, managed activities such as specific carbon removal technologies.
No abstract	Records without an abstract are considered non-relevant.

For the full screening we employed the machine learning tool ASReviewer to assist with the screening process. The tool presents records consisting of a title and abstract to the researcher, who classifies them using a binary label (1 for relevant, 0 for irrelevant). These labels are then used to train the model, and the tool subsequently presents additional records for classification. This cycle continues until a user-defined stopping criterion is met, allowing the screening to stop before all items have been reviewed by the researcher. The tool was selected for its open-source nature, well-documented use instructions, and previous applications in peer-reviewed systematic literature reviews [9], which support the goals of reproducibility and transparency in our own review process. A detailed description of the tool and its application can be found in van de Schoot et al [8]. As ASReviewer is an open-source tool, it should be accessible to anyone wishing to apply the methodology. If another machine-learning tool is used, it should likewise employ an active-learning, human-in-the-loop approach, with comparable learning procedures for the model, extraction strategy, and stopping rule. A comparison with other machine-learning or artificial intelligence (AI) tools is beyond the scope of this method.

For model configuration, we used the tool’s default settings as best practice, which include a Naive Bayes classifier, Term Frequency-Inverse Document Frequency (TF-IDF) feature extraction, and a dynamic resampling balance strategy [9,10,8]. For the stopping rule, we adopted the approach developed by Callaghan and Müller [11] for both the carbon and biodiversity datasets. This rule utilizes a flexible statistical stopping criterion, which rejects the hypothesis of having missed a specified recall target with a predefined level of confidence [11]. For our review, we set the recall target at 90 %, based on reported error rates of 10–13 % in manual reviews [12,13], with a significance level of *p* < 0.05, achieving *p* = 0.046 for both biodiversity and carbon respectively. Further quantitative documentation of this step is included in the PRISMA flow chart in Fig. 1.

However, for the water dataset, we needed to adjust the stopping rule. Given the large volume of records obtained from the databases (*n* = 1753 after deduplication) and prior knowledge suggesting that only a small subset would be relevant (<50), applying the stopping criteria outlined by Callaghan and Müller [11] would have required an excessive number of iterations with non-relevant items. We implemented an alternative stopping rule tailored to the dataset’s specific characteristics, adopting a modified stopping rule based on the SAFE procedure outlined by Boetje and van de Schoots [14]. The stopping rule determined to stop after 200 consecutive non-relevant items, after at least 10 % of the total records had been screened, and once all previously identified relevant publications were found.

## Analysis

**Fig. 2:**
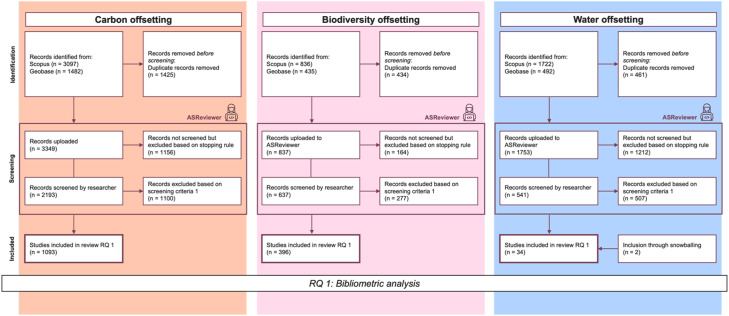
Excerpt PRISMA flowchart bibliometric analysis.

**Fig. 3:**
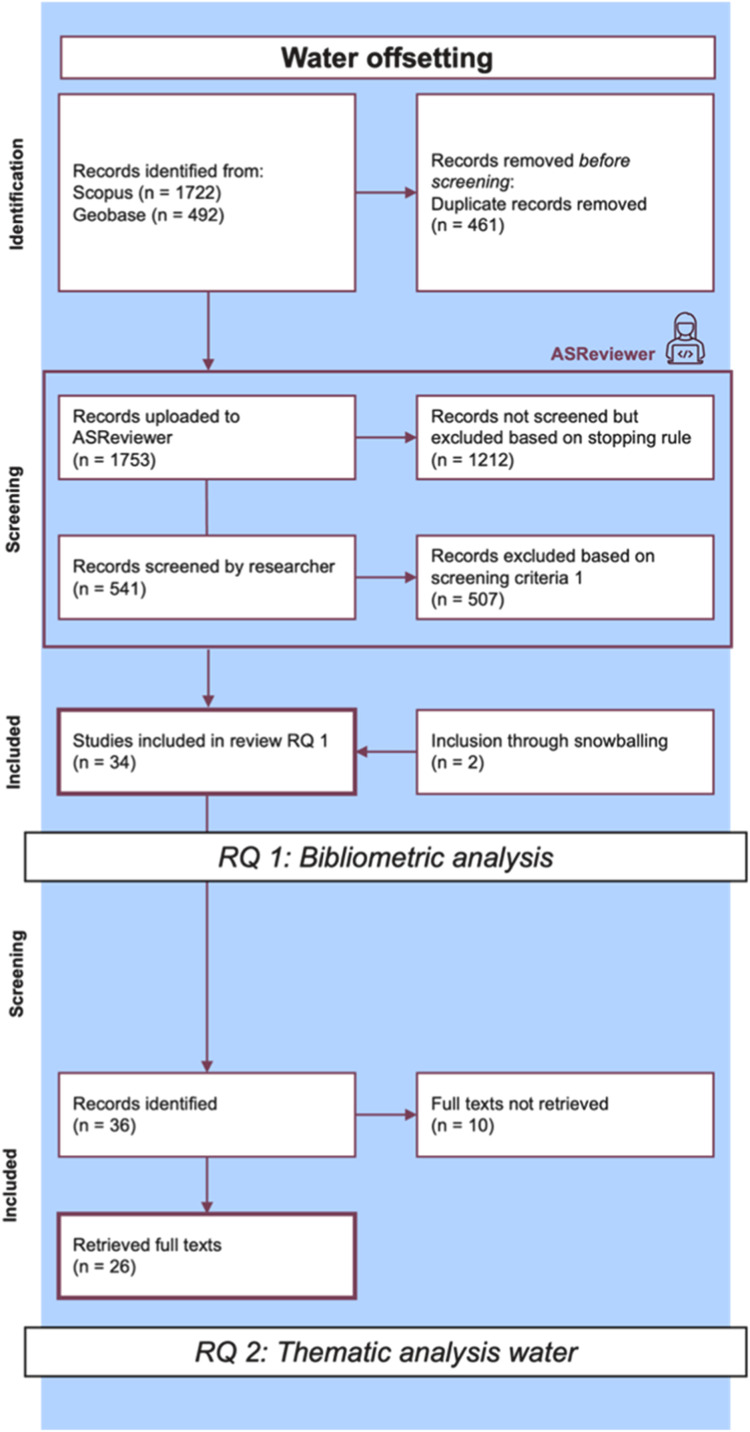
Excerpt PRISMA flowchart thematic analysis water.

**Fig. 4:**
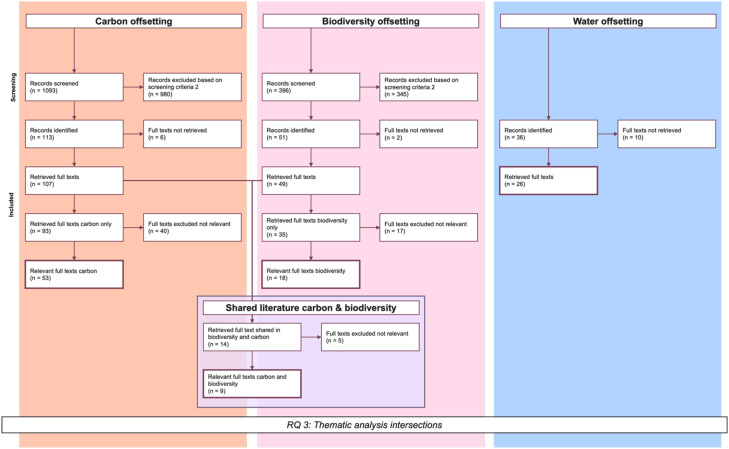
Excerpt PRISMA flowchart thematic analysis intersection.

The results were analysed in three steps, combining quantitative and qualitative approaches. This allowed for a comprehensive understanding and comparison of the datasets, as well as a detailed introduction to the water offsetting literature. While we present the methodological approach to the analysis here, the results and discussion of the analysis itself are provided in the related research article (related research article). The datasets used at each step of the analysis are illustrated in Fig. 1 (the PRISMA flowchart). Excerpts of the flowchart relevant to each step of the analysis are shown in Fig. 2, Fig. 3, and Fig. 4, respectively.

### Bibliometric analysis

The resulting three datasets of relevant literature (see Fig. 2) were used for the bibliometric analysis, which was conducted using the R package bibliometrix and its web-based interface, biblioshiny [15]. The analysis was performed individually for each dataset and included basic descriptive statistics, annual scientific production, country-specific contributions, publication sources, keyword co-occurrence analysis, and trend topic analysis. The results of these analyses were compared across the datasets and visualized using the same R package and are presented in the original publication (related research article).

### Qualitative analysis of the water literature

This section of the analysis includes only the relevant full-text items from the water dataset (see Fig. 3), identified through ASReviewer, along with two additional items identified as references cited within the relevant items (snowball sampling). It provides a detailed introduction to water offsetting and water neutrality as emerging concepts. For the analysis, a thematic analysis approach (following Braun and Clarke [16] and Pan et al [17]) was employed using the qualitative data analysis software Atlas.ti. The initial coding framework was based on prior knowledge from the carbon and biodiversity literature and the relevant concepts identified within those fields. The code system was continuously refined during the coding process and re-applied accordingly. The results were then summarized and compared with definitions from the carbon and biodiversity literature. Additionally, mentions of biodiversity or carbon were coded during this step, offering a comprehensive understanding of how the water literature engages with these topics. An adjusted approach to analyse the intersection for the other two datasets is presented in the next step, as the results contribute to addressing the subsequent part of the research. All findings can be found in the original research article (related research article).

### Qualitative analysis of the intersection

This section of the analysis incorporates the results from the previous step on the intersection of water with carbon and biodiversity. For the remaining analysis, the relevant items from the carbon and biodiversity datasets were used. Given that carbon resulted in 1093 relevant items and biodiversity in 396 (see Fig. 4), conducting a comprehensive full-text analysis of all items was beyond the scope of this review. Instead, we focused on the intersection between the three offsetting types.

To achieve this, a second screening step was applied, where keywords and abstracts of the relevant carbon and biodiversity items were searched for mentions of the other two offsetting types (screening criteria 2). From these, we also identified items that were part of both the carbon and biodiversity datasets. This led to the creation of three distinct groups: (1) carbon literature with mentions of biodiversity and/or water, (2) biodiversity literature with mentions of carbon and/or water, and (3) literature identified in both carbon and biodiversity datasets that includes mentions of carbon, biodiversity, and/or water. These datasets were then reduced based on the availability of full texts and their actual relevance, as determined during the qualitative analysis. Relevance was assessed based on the focus on the intersection; therefore, the text needed to address the intersection between at least two offsetting types in some way.

For each group, full texts were identified, and a thematic analysis approach again following Braun and Clarke [16] and Pan et al [17] was applied using the qualitative data analysis software Atlas.ti. The initial code structure, based on prior knowledge and previous coding of the water literature, was expanded and reapplied throughout the coding process. Due to the still large body of literature, this analysis did not involve a full-text review of all items. Instead, search terms were repeatedly used to identify relevant text passages, and the coding was applied to these identified sections. The full results of this analysis can be found in the original research paper (related research article).

### Limitations

The limitations of this approach are typical of many literature reviews. Firstly, it primarily relied on English-language publications from dominant Western databases, potentially overlooking important non-Western perspectives on offsetting. This gap could be addressed in future research by including additional languages and incorporating non-Western databases, or only using non-Western databases. The search terms used may have excluded relevant studies due to variations in terminology or unconventional keywords, thereby potentially introducing bias. To mitigate this risk, the search strings were discussed within an interdisciplinary team and refined based on feedback from an information specialist. Although the ASReview machine learning tool helped manage the large volume of studies, there remains the possibility that relevant articles were excluded or irrelevant ones included, due to human error and the residual risk from non-manually screened records. Grey literature was excluded given the scope of the review, but it might contain valuable insights or data.

The specific limitations of this adaptation include the increased time and resources required to perform multiple scoping reviews. While the method is inherently interdisciplinary, the choice of database and inclusion criteria may influence the disciplinary coverage and therefore needs to be carefully considered to account for differences between natural and social sciences. The challenge of making these reviews comparable arose from the need to apply the same approach while accounting for the unique characteristics of each dataset. As a result, only a limited focus could be analyzed, requiring a balance between providing a holistic analysis and maintaining a specific focus. While harmonizing the screening criteria improved consistency and helped reduce bias during the screening process, the large and diverse body of literature made uniform decision-making challenging, particularly for ambiguous cases. The qualitative coding process also introduced some subjectivity, as it relied on the researchers' interpretations.

These limitations should be considered when adapting this approach and interpreting the findings.

### Ethics statements

This is an adaptation of a scoping review, so there is no involvement of patients or the public. Ethical approval is not required, as the review is based on previously collected data.

## CRediT authorship contribution statement

**Felice Diekel:** Conceptualization, Methodology, Investigation, Formal analysis, Writing – original draft, Visualization. **Rosalie Arendt:** Conceptualization, Writing – review & editing, Supervision. **Markus Berger:** Writing – review & editing, Supervision.

## Declaration of competing interest

The authors declare that they have no known competing financial interests or personal relationships that could have appeared to influence the work reported in this paper.

## Data Availability

No data was used for the research described in the article.
